# Impact of psychosocial stress on facial emotion recognition in schizophrenia and controls: an experimental study in a forensic sample

**DOI:** 10.3389/fpsyt.2024.1358291

**Published:** 2024-07-16

**Authors:** Henning Hachtel, Gunnar Deuring, Marc Graf, Tobias Vogel

**Affiliations:** ^1^ Forensic Department, University Psychiatric Clinic Basel, Basel, Switzerland; ^2^ Medical Faculty, University of Basel, Basel, Switzerland

**Keywords:** psychosocial stress task, subjective stress response, physiological stress response, arithmetic task, naming and comparing facial emotion tasks

## Abstract

**Introduction:**

Psychotic disorders have been associated with dysregulated stress reactions and adaptation. Little is known about the neuroendocrine responses to psychosocial stress in justice-involved individuals with schizophrenia.

**Methods:**

Using an experimental research design, the present study aims to examine differences in the subjective and neuroendocrine responses to psychosocial stress and its impact on facial emotion recognition (FER) and performance on an arithmetic task in chronically ill justice-involved individuals with schizophrenia (PAT) and a healthy control group. PAT undergoing treatment in forensic psychiatric inpatient wards (n = 17) and a healthy control group (n = 17) were assessed regarding sociodemographic and clinical characteristics. Additionally, salivary cortisol levels, measured before and after performing a psychosocial stress task [Montreal Imaging Stress Task (MIST)], and performance on an arithmetic problem-solving task and two FER tasks were recorded. Two participants dropped out, one from each group. Therefore, the final sample consisted of 32 individuals.

**Results:**

Significant group differences in FER were recorded. There was a significant rise in subjective perception of momentary strain relating to the induction of psychosocial stress in both groups. Notably, the pre-stress level of subjective strain was higher in the PAT group than controls. Acute psychosocial stress induced an increase in FER performance in a sub-task related to naming emotions in individuals with schizophrenia spectrum disorder.

**Discussion:**

The results underline the importance of psychosocial and therapeutic interventions aimed at strengthening stress resilience in individuals with schizophrenia spectrum disorders.

## 1 Introduction

Psychosocial stress has been defined as any social or cultural situation that causes physical, emotional, or psychological strain on an individual (1). The evaluation of psychosocial stress and its reactions has attracted some interest in the scientific literature (2). Inducing stress in an experimental setting is often used as a valid proxy for real-life stressors, leading to subjective and neuroendocrine responses to stress, which can be measured using cortisol levels and heart rate (3). It is well established that exposure to stress can trigger neuroendocrine responses involving the endocrine and autonomic nervous systems (4). Indeed, psychosocial stress is one of the strongest factors that can increase the activity of the hypothalamic–pituitary–adrenal (HPA) axis, a major neuroendocrine system that controls reactions to stress (5).

Environmental factors such as exposure to psychosocial stress and childhood trauma have been implicated in the aetiology of psychotic disorders (6). Psychotic disorders have been associated with dysregulated stress reactions and adaptation. Individuals with schizophrenia are generally more susceptible to stress, show differences in stress processing (7–9), and have a blunted cortisol reactivity compared with controls (10). Subjective responses to psychosocial stress seem to be related to the course of the disorder. While individuals with first-episode schizophrenia are reported to be more disposed to higher stress reactions, those with chronic forms of schizophrenia do not differ in their stress reactions when compared with healthy controls (11). Furthermore, stress is regarded as an important factor that disturbs cognitive, affective, and perceptive processes (12). Although existing evidence suggests that social and non-social cognitions are largely distinct, both cognition types share overlapping processes such as working memory and perception (13). Previous literature reports substantial impairments in schizophrenia, not only in these facets of cognition but also in cognitive control, attention, and processing speed (14).

The study of social cognition (i.e., cognitive processes and behaviours, which underlie human social interactions) often relies on information-processing paradigms such as facial emotion recognition (FER) tasks (15). FER plays a vital role in guiding social interactions (16). Although both FER and empathy involve understanding the emotions of others, they are distinct constructs since FER only encompasses the perceptual ability to categorise faces according to their emotional expressions (16). FER can be evaluated using non-behavioural (e.g., MRI and electroencephalography) and behavioural tools such as FER tasks (17).

Previous studies have consistently reported deficits in facial emotion recognition in people with schizophrenia relating to impaired cue processing in social cognition (18, 19). Research suggests that impairment in FER precedes the onset of schizophrenia and remains a constant feature over the course of the illness (20). Additionally, impairment in FER is reported to increase with the number of psychotic episodes over the course of schizophrenia (21). It is reported that childhood trauma and parental bonding can influence the ability of FER even in healthy individuals (22, 23). Difficulties in integrating social context information are further reported to be associated with impairments in FER in people with schizophrenia (24).

There has been a growing interest in understanding the impact of psychosocial stress on functioning and FER in healthy subjects and individuals with schizophrenia (25). A systematic review reported that patients with first-episode psychosis had higher subjective responses to stress and lower stress-induced cortisol levels than controls (11). Other studies reported inconsistent findings regarding the impact of acute psychosocial stress on emotion recognition, with some reporting an improvement in emotion recognition in healthy individuals under psychosocial stress (25). Dysfunctions in FER, the impact of stress on mental health, poor coping skills, and social functioning have further implications for the risk of violence (26–28), although some studies reported similar distributions of FER misidentifications among violent and non-violent individuals with schizophrenia (29). Despite the importance of these observations, there is a dearth of studies examining the relationship between exposure to stress and FER in forensic populations (30, 31). To date, no published studies examined the impact of acute psychosocial stress on FER in forensic populations with schizophrenia.

### 1.1 Study aims

Using an experimental research design, this study aims to compare i) the subjective experiences (e.g., self-assessed momentary strain) and neuroendocrine (e.g., salivary cortisol level) responses to psychosocial stress, ii) the impact of psychosocial stress on FER, and iii) overall performance on an arithmetic task (AT) in individuals with chronic schizophrenia who are under the care of a forensic mental health service and in healthy controls.

### 1.2 Hypotheses

We hypothesised that there would be no significant group differences in subjective responses to stress but that individuals with schizophrenia will exhibit impaired physiological responses to stress and impaired performance on arithmetic and FER tasks compared to control. We also hypothesised that FER performance will be aggravated by the induction of psychosocial stress in both groups.

## 2 Methods

### 2.1 Design and participants

To control for the potential confounding effects of sex on facial emotion recognition (32, 33), a male-only sample comprising 34 subjects was included in the study: 17 inpatients with schizophrenia (*PAT* group) from the forensic psychiatric clinics of Basel (UPK Basel) and Königsfelden (PDAG Königsfelden) in Switzerland and 17 age- and education-matched controls (*CTL* group) recruited through advertisements.

### 2.2 Procedure

*CTL* was screened using Structured Clinical Interview for Diagnostic and Statistical Manual of Mental Disorders (DSM) (SCID) I and II (34) to ascertain the absence of psychiatric disorders. Recreational drug use in *CTL* was not an exclusion criterion, but individuals with a diagnosis of a substance use disorder, which incorporates harmful use and more severe dependency, were not eligible for inclusion in the study. *PAT* were under supervision in inpatient settings where substance use is strictly controlled. This ensured that all individuals in this group remained abstinent from substances before and during the study. To minimise bias related to prior knowledge of the impact of psychosocial stress, participants were informed that the primary focus of the study was on measuring performance on arithmetic and FER tasks while pointing out that stress could be induced as part of the process. Two participants dropped out, one from each group. Therefore, the final sample comprised 32 participants, 16 in each group (see also the Statistical Analysis section).

Assessment of participants’ sociodemographic and clinical characteristics was undertaken at baseline, followed by a separate session lasting approximately 130 min for measuring FER, AT performance, and stress responses. This session started consistently at 2 p.m. to ensure similar conditions with respect to participants’ diurnal salivary cortisol levels.

Although wake-up times were not explicitly recorded in this study, it is worth noting that in the inpatient setting of *PAT*, bedtimes were regulated and wake-ups did not occur later than 7 a.m. For *CTL*, this information was not collected, but individual baseline differences were effectively controlled for in the linear mixed model method by putting emphasis on the observation of changes in salivary cortisol through stress induction among groups. The study paradigm was designed to capture as much information as possible from participants, including the administration of study questionnaires before the induction of psychosocial stress to lower the chances of dropouts relating to exhaustion and/or low motivation. The Childhood Trauma Questionnaire (CTQ; 35) and Parental Bonding Inventory (PBI; 36) were also included to ensure that the groups did not differ with respect to these potential confounders. The two FER tasks were administered before and after the induction of stress. An outline of the whole procedure is given in **Table 1**.

**Table 1:** Procedures.

Session day 1: Assessment of participant characteristics.		
• Sociodemographic data, including handedness • IQ: MWT-A (Mehrfachwahl-Wortschatz Test) • Clinical symptoms and psychopathology: Brief Psychiatric Rating Scale (BPRS) • Positive and Negative Syndrome Scale (PANSS) • Childhood Trauma Questionnaire (CTQ) • Parental Bonding Inventory (PBI) •		

Session day 2: Assessment of facial emotion recognition (FER), arithmetic task (AT) performance, and stress response (CORT and KAB)		
• Introduction, measurement of body weight and length, and sensor application		
**Point of measurement**	**Duration**	**Procedure**
*Pre 1*	2 min	CORT and KAB
	21 min	Sitting quietly, RAPA questionnaire, reading a magazine
*Pre 2*	2 min	CORT and KAB (*pre*-stress for KAB)
	**16 min**	**FER tasks A and B**
*Pre 3*	2 min	CORT and KAB (*pre*-stress for cortisol)
	**26 min**	**MIST social stress induction and AT assessment**
*Post 1*	2 min	CORT and KAB (*post*-stress for KAB)
	**12 min**	**FER tasks A and B**
*Post 2*	2 min	CORT and KAB (*post*-stress for cortisol)
	18 min	Sitting quietly, reading a magazine
*Post 3*	2 min	CORT and KAB
	18 min	Sitting quietly, reading a magazine
*Post 4*	2 min	CORT and KAB
• End of session		

### 2.3 Exclusion criteria

Exclusion criteria for the *CTL* group were the diagnosis of major psychiatric disorders such as psychotic disorders, affective disorders, substance use disorders, intellectual disability, or personality disorders; a history of traumatic brain injury; severe neurological disorders (e.g., Parkinson’s and epilepsy); continuous benzodiazepine medication; and severe language delay or poor German comprehension.

### 2.4 Ethics approval and informed consent

The study was approved by the Ethics Committee of Northwestern and Central Switzerland (EKNZ Reference number 2016–00701). All participants provided written informed consent.

### 2.5 Assessment of participant sociodemographic and clinical characteristics

After confirming diagnoses using SCID-I and SCID-II (37, 38), information about sociodemographic characteristics (including handedness) was obtained from patients’ files or, in the case of the control group, the interview. The *PAT* group was strictly monitored regarding medical treatment (including drug monitoring) and use of psychotropic substances including PRN medications, and relevant information was obtained from clinical files. Current IQ was assessed using the MWT-A (Mehrfachwahl-Wortschatz Test; 39).

Clinical symptoms and psychopathology were assessed using the Brief Psychiatric Rating Scale (BPRS; 40) and the Positive and Negative Syndrome Scale (PANSS; 41). Overall levels of functioning were measured using the Global Assessment of Functioning (GAF) scale (42). The CTQ (35) and PBI (36) were utilised to assess childhood victimisation.

### 2.6 Assessment of facial emotion recognition

**Figure 1:**
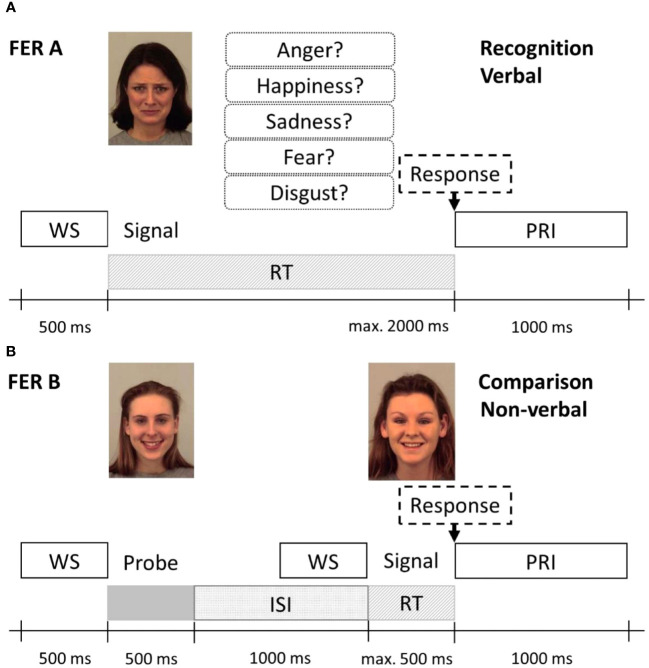
Protocol schemes of the Facial Emotion Recognition Tasks FER A **(A)** and FER B **(B)**. **(A)** In the FER A verbal recognition task, the emotional face signal and the response options were shown simultaneously until a response was selected or the time limit of 2,000 ms was reached. **(B)** In the non-verbal emotion comparison task FER B, first, the probe (left) was shown, followed by the signal (right), which was either a corresponding or non-corresponding emotion (here, a corresponding emotion is presented). The response time limit here was 500 ms. WS, warning signal; ISi, inter-stimulus interval; PRI, post-response interval. Stimulus images from the Karolinska Directed Emotional Faces set, IDs: AF01SAS, AF05HAS, AF09HAS.

To assess the ability to recognise emotions from facial expressions, we used two specific FER tasks based on Comparelli et al. (20) (subtest FER A: naming task/verbal modality) and Barkhof, de Sonneville, Meijer, and de Haan (43) (subtest FER B: recognition task/non-verbal modality) for recognition of five basic emotion expressions: happiness, sadness, anger, fear, and disgust. Signals consisted of 162 pictures out of the Karolinska standardized picture set (44), a complete list of the used images can be found in the **Supplementary Material**. In subtest FER A, a randomly selected image of facial expression was shown on a computer screen together with a panel assigning the names of the five emotions to a number (see **Figure 1A**). Participants were asked to indicate the recognised emotion by entering the corresponding number via a computer keyboard. Each emotion occurred seven times (7 × 5 = 35 trials), and the time limit for responding was 2,000 ms. Correctly recognised emotions were scored as 1, yielding a total score of 0–35. In subtest FER B (see **Figure 1B**), participants were asked whether two sequentially presented faces were showing the same emotion or not by pressing the corresponding key on the keyboard within 500 ms. This task examines the ability to properly identify facial emotion expressions by comparing these to different facial emotion expressions of another person without the need to express this verbally. FER B, therefore, examines facial emotion recognition without the additional task of verbal labelling. A total of 40 trials were conducted comprising 20 matching trials (“yes condition”) and 20 non-matching trials (“no condition”) in random succession. Both faces in a trial were of the same sex; emotion and sex were evenly distributed across the test run. Correct responses were scored; the possible total score was 0–40 (and 0–8 for each emotion).

### 2.7 Assessment of arithmetic task performance and stress response

The Montreal Imaging Stress Task (MIST; 45) was used to induce moderate psychosocial stress and measure AT performance. The MIST consisted of a series of computerised mental arithmetic challenges that were presented on a computer screen, and subjects had to submit their answers by means of a response interface (keyboard) within a variable time limit. Task difficulty and time limit were manipulated to be just beyond an individual’s mental capacity so that the rate of correct responses would never exceed 50%. Information on individual current performance as well as the expected performance was indicated on screen. To further increase the social evaluative threat of the situation, direct negative feedback on the subjects’ task performance was given between each of the three separate runs by an increasing number of investigators and their expressed urgency to perform better. The accuracy (hit rate) of the ATs was recorded at each run set and used as the outcome measure.

Salivary cortisol (CORT) as a measure of physiological stress response was sampled at three time points before (*pre 1–3*) and four time points after stress induction (*post 1–4*). Cortisol concentrations were quantified using a high-sensitivity salivary cortisol enzyme immunoassay kit (Salimetrics Europe, London, UK), following the manufacturer’s protocol (**Supplementary Material for details**). Concurrently, the Kurzfragebogen zur Aktuellen Beanspruchung (KAB; 46) was administered to assess the subjective stress response (see **Table 1**, Session day 2). The KAB was designed to evaluate the strain of subjective psychosocial stress in self-report form within short periods of time (i.e., 0.5 min). It consists of six items measured on a 6-point Likert scale including dimensions of stress (e.g., I feel worried and I feel sceptical) and relaxation (e.g., I feel relaxed and I feel detached). In the evaluation, half of the items were reversed so that higher strain was expressed through higher scores.

### 2.8 Statistical analysis

For normally distributed data, Student’s t-test for independent samples was applied for univariate group comparisons of demographic and psychological test and questionnaire variables. Otherwise, the non-parametric Wilcoxon rank-sum test was used.

For the analysis of the effect of stress induction on salivary cortisol, FER A, and FER B among the study groups a linear mixed model (LMM) approach was employed. The LMMs for all outcome variables had the same factorial structure: a within-subject factor *Stress* with two levels, *pre* and *post* stress induction, and a between-subject grouping factor *Group* with two levels, *PAT* and *CTL*. Non-significant terms were eliminated stepwise from the fully crossed two-factorial model, and only significant simple main effects were reported and interpreted.

The univariate group comparison of arithmetic task performance was tested using a one-way ANOVA.

Due to sampling errors and subject’s incompliant responses, the number of eligible subjects and available data points varied for each analysis. A value of *p* <.05 was set to indicate statistical significance.

All statistical procedures were carried out using the R statistics environment version 4.0.3 (47). For the LMM modelling, lmerTest v3.0–3 (48) and lme4 v1.1-27–1 (49) statistical packages were used.

Evaluation of performance and model diagnostics was conducted using the package performance (v0.7.0; 50). Fixed LMM effects were tested by one- or two-way analysis of deviance (AoD), which performs Wald-type chi-squared (Χ^2^) statistics. Cohen’s omega squared (ω^2^) is provided as an effect size measure for the LMM fixed effects (package effectsize v0.4.5; 51). The interpretation was as follows: small effect: ω^2^ ≥.01, medium effect: ω^2^ ≥.06, and large effect: ω^2^ ≥.14, comparable to eta-squared (52). Partial omega squared (ωp^2^) was reported for multiple fixed-effect models. Significant AoD fixed effects were tested post-hoc for significant group differences (package emmeans v1.5.4; Lenth, 2021). Hedges’ g was computed as effect size for post-hoc contrasts (small effect: g ≥.20, medium effect: g ≥.50, and large effect: g ≥.80, comparable to Cohen’s d; 52). Dependent variables not meeting the assumptions of homoscedasticity and heterogeneity of variances were submitted to a Box–Cox power transformation prior to analysis (package MASS v7.3–50; 53); figures depict response scale values. LMMs presenting yet unmet assumptions were further analysed using a CR2 adjusted, cluster-robust variance–covariance matrix (package clubSandwich v0.5.3; 54).

An a priori power analysis to determine the sample size was performed using the G-Power application for Microsoft Windows; for details, see the Power Analysis section in the **Supplementary Material**.

## 3 Results

### 3.1 Sample characteristics

Seventeen patients and 17 age- and education-matched control subjects were enrolled in the study. One participant in each group dropped out, resulting in a combined sample of 32; see group statistics in **Table 2**. The groups were largely similar, except for mean verbal IQ, which was significantly higher for the CTL group (*p* = .02). Of note, no significant differences in dimensions of childhood trauma or styles of parental bonding were observed in the two groups (all *p* ≥.05), which averted the necessity to include these potential confounders in the statistical analyses and consequently increase the sample size to maintain adequate power. An accurate investigation of the confounding effects was not intended. The level of psychopathology in the *PAT* group assessed by the PANSS was low (see **Table 2**). The mean daily dose equivalent of the antipsychotic medication (55) was chlorpromazine 618 mg (*SD* = 438 mg). The distribution of four categories of recorded offenses among the 16 subjects in the CTL group was as follows: 7 had no offense (43.75%), 3 were convicted of [1] (18.75%), also 3 committed [3] (18.75%), 2 were [4] offenders (12.50%), and lastly 1 had a record of [2] (6.25%).

**Table 2:** Demographics and sample characteristics by group.

	Patients		Controls		
	*n* = 16		*n* = 16		
	Mean	*SD*	Mean	*SD*	p*
Age (years)	33.2	8.0	32.8	10.6	0.91
Education (years)	11.7	2.5	12.5	2.1	0.34
Illness duration (years)	11.6	7.9			
PANSS—positive scale	12	4.0			
PANSS—negative scale	15.9	5.2			
Chlorpromazine equivalent (mg)	618	438			
PANSS—general psychopathology	24.3	4.1			
BPRS	31.5	4.5			
CTQ total score	38.0	6.9	40.5	16.7	0.55
CTQ emotional abuse	7.1	3.1	9.1	4.3	0.05
CTQ physical abuse	5.8	1.3	7.0	5.1	0.81
CTQ sexual abuse	5.9	2.2	5.2	0.5	0.55
CTQ emotional neglect	11.6	4.2	11.3	5.5	0.43
CTQ physical neglect	7.7	2.2	7.9	3.5	0.77
Verbal IQ	102.8	17.6	115.1	11.3	0.02
PBI mother care	23.4	6.8	24.1	8.3	0.61
PBI mother overprotection	11.4	5.0	11.1	7.8	0.37
PBI father care	22.9 ^1^	7.0	21.7	7.0	0.65
PBI father overprotection	10.4 ^1^	5.2	8.3	3.7	0.21

SD, standard deviation; PANSS, Positive and Negative Syndrome Scale; BPRS, Brief Psychiatric Rating Scale; CTQ, Childhood Trauma Questionnaire; PBI, Parental Bonding Inventory.

^1^n = 14, 2x no father;

^*^Student’s t-test or Wilcoxon test was applied for group comparisons.

Details on other analyses, i.e., AoDs, contrasts, and estimated marginal means, are provided in **Supplementary Material Tables S1**-**S3**.

### 3.2 Stress response

#### 3.2.1 Salivary cortisol

**Figure 2:**
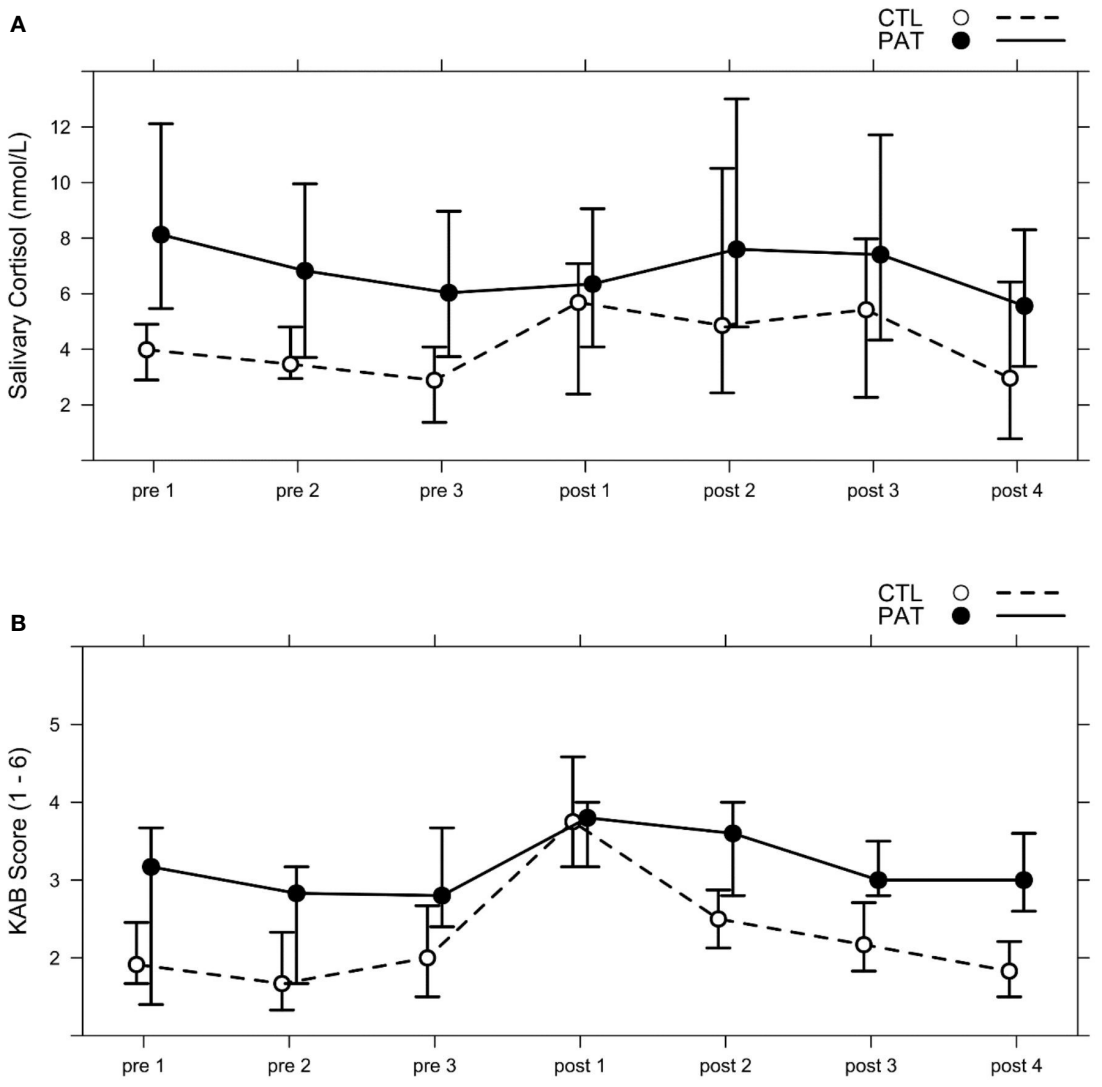
Median progression of **(A)** salivary cortisol levels (nmol/L) and **(B)** subjective momentary strain measures (1–6) in both groups (schizophrenic patients, *PAT*; healthy controls, *CTL*) across the seven points of measurement (*pre 1–3*; *post 1–4*); see **Table 1**, Session day 2. The social stress paradigm MIST was applied between *pre 3* and *post 1*, and the emotion recognition tests FER A and FER B were both administered after *pre 2* and after *post 1*. Whiskers show percentages of 25% and 75%.

The average progression of salivary cortisol levels for each group is shown in **Figure 2A**. Across all subjects, cortisol level was the lowest immediately before stress induction at *pre 3* and peaked at *post 2*, approximately 35 min after the social stress induction onset. These two sampling points defined the two levels (*pre* and *post*) of the within-subject factor *Stress*. Prior to analysis, cortisol data were Box–Cox power-transformed (λ = 0.20), and two subjects were excluded due to missing samples.

**Figure 3:**
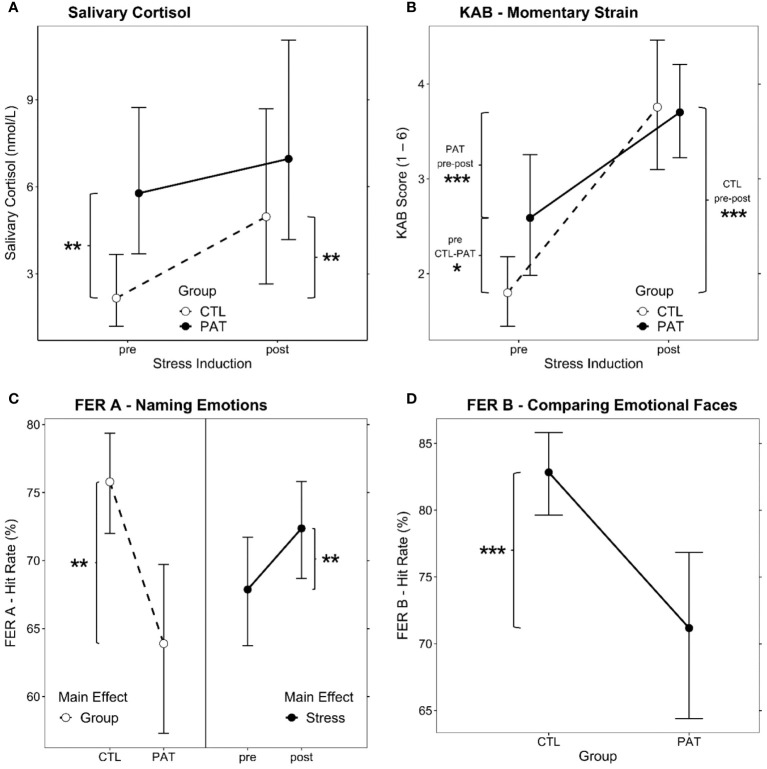
Estimated marginal means plots of the significant LMM fixed effects. *Group* × *Stress* interaction effect for **(A)** salivary cortisol levels and **(B)** KAB self-rating of momentary strain. **(C)** Main effects for *Group* (left, both points of measurement combined) and *Stress* (right, both groups combined) for hit rate in the emotional faces recognition task FER **(A, D)** *Group* main effect for hit rate comparing emotional facial expressions, FER B (both points of measurement combined). LMM, linear mixed model; PAT, schizophrenic patients; CTL, healthy controls; pre/post, before/after stress induction. Brackets show significant *post-hoc* comparisons (simple effects), and significance levels are indicated as *p <.05, **p <.01, and ***p <.001. Error bars show 95% confidence intervals for the estimated marginal means.

The AoD of the LMM revealed a significant interaction between the fixed effects *Group* and *Stress* [Χ^2^_(1)_ = 4.90, *p* = .03, ω_p_^2^ = 0.13] (see **Figure 3A**). *PAT* showed higher baseline cortisol levels prior to stress induction than *CTL* [*pre CTL* vs*. PAT*: *T*_(40)_ = 3.70, *p* = .001, *g* = 1.32]. Stress induction increased salivary cortisol levels significantly in the *CTL* group [*CTL pre* vs*. post*: *T*_(26)_ = 3.71, *p* = .001, *g* = 0.97], but not in patients. Maximum post-stress levels among both groups were similar [*post* CTL vs. PAT: *T*_(39)_ = 1.16, *p* = .25].

#### 3.2.2 KAB rating of subjective momentary strain

Subjective momentary strain was rated by subjects seven times parallel to the saliva cortisol sampling framework (see **Figure 2B**). The average KAB scores across both groups were at minimum *pre 2*, before the beginning of the pre-stress FER tasks, and at maximum at *post 1*, immediately after the MIST stress induction block. The transformed KAB scores (λ = 0.6) of these two points of measurement were used to assess subjective strain responses to stress in both groups.

AoD yielded a significant *Group* × *Stress* interaction effect [Χ^2^_(1)_ = 7.30, *p* = .007, ω_p_^2^ = 0.16] (see **Figure 3B**). Both groups reported higher KAB ratings after the stress treatment than before [*CTL pre* vs*. post*: *T*_(30)_ = 9.30, *p* <.001, *g* = 2.35; *PAT pre* vs*. post*: *T*_(30)_ = 4.01, *p* <.001, *g* = 1.19]. However, where *PAT* presented with significantly higher strain ratings before stress induction [*pre CTL* vs*. PAT*: *T*_(54)_ = 2.85, *p* = .006, *g* = 0.98], the reported post-stress level was not different between groups [*post CTL* vs*. PAT*: *T*_(54)_ = 0.17, *p* = .87].

### 3.3 FER A and B, naming and comparing facial emotions

Hit rate values of the FER A paradigm were Box–Cox power-transformed with λ = 2.28. One subject was excluded due to extremely low hit rates of ≤0.2.

Both main effects of the LMM showed significance [*Group*: Χ^2^_(1)_ = 12.58, *p* <.001, ω_p_^2^ = 0.26; *Stress*: Χ^2^_(1)_ = 7.26, *p* = .007, ω_p_^2^ = 0.16]; no interaction was present. As is apparent from the left-hand side of **Figure 3C**, overall, patients performed worse than controls, and stress induction brought about a similar significant performance increase in facial emotional expression recognition in both groups (right-hand side of **Figure 3C**).

In order to evaluate the potential effects of training during the first FER A test run on the pre- vs. post-stress performance, the linear slopes of individual learning curves in the pre-condition were derived and tested against zero for each group by one-sample t-tests. In both groups, the mean learning slope was not distinct from zero (all *p* >.18), which indicates that the influence of an assumed training effect on the observed pre–post stress result is negligible. Furthermore, when introducing the learning slope as a covariate into the model, it showed a significant association with hit rate [Χ^2^_(1)_ = 4.95, *p* = .03], but no interaction with the two factors *Group* or *Stress*, which underlines the assumption that training effects are also not contributing to group differences.

Also, for the emotion comparison paradigm FER B, group differences and the effect of stress on the hit rates (Box–Cox power-transformed; λ = 3.03) were evaluated by AoD. Only the *Group* effect was significant [Χ^2^_(1)_ = 14.12, *p* <.001, ω^2^ = 0.28] (see **Figure 3D**), pointing out the patient’s overall deficit in that task. Stress induction had no effect on the FER B performance in any group.

### 3.4 Arithmetic task performance

Subjects’ average performance in the arithmetic tasks, i.e., the proportion of correct responses (*hit rate*), was employed as a surrogate measure of cognitive abilities that are independent of social context. One-way ANOVA of the *Group* difference revealed that patients performed significantly worse in the AT than controls [*F*_(1,30)_ = 11.58, *p* = .002, ω^2^ = 0.25].

## 4 Discussion

This study is the first of its kind to examine the impact of acute psychosocial stress on FER in a sample of justice-involved individuals with schizophrenia in comparison to a control group. The extant literature (e.g., 56) supports a positive link between mental disorders like autism spectrum disorders and impairments in FER. In our study, participants were adequately assessed, and no comorbidities with known associations with impairments in FER were detected. As the control sample predominantly had a history of offences as well as the whole *PAT* group, we considered that differences in FER do not necessarily reflect criminal tendencies at a group level but are related to the pathophysiology of schizophrenia. There is evidence to support the link between childhood trauma and lower scores on parental bonding (23). This holds true for people with schizophrenia. For example, a study (57) involving participants with major mental disorders recruited from outpatient centres found that only one-third of patients with schizophrenia experienced an optimal parental bonding style (low control and high care), which was also the most prevalent style of parental bonding. At the same time, almost two-thirds of the participants in this study reported inefficient paternal bonding styles and approximately half from inefficient maternal bonding styles.

Surprisingly, our sample did not differ significantly in the quality of parental bonding. We could not substantiate if this was due to recall bias, inability to recall due to memory suppression, other psychodynamic defence mechanisms, sampling bias, or missing differences. However, it is likely that the *CTL* is not representative of the general population. We reproduced well-established findings in the literature, which highlighted deficits in facial emotion recognition in people with schizophrenia spectrum disorders (58, 59). In our study, childhood trauma and parental bonding did not significantly differ between the groups, and as such, no inference can be made that parenting style influenced FER decisively. As deficits in attention, working memory, episodic memory, processing speed, and executive functions in schizophrenia are broadly documented (60), we assumed that an increase in psychosocial stress would also cause a significant decrease in FER. Our hypothesis was not supported by the results of the naming task, which showed an increase in task performance after the induction of psychosocial stress and no non-significant changes in performance in the comparison task in both groups. Recent literature reported a significant increase in emotion detection performance and significantly shorter response latencies under acute stress, independent of emotional valence or emotion intensity (61). Our results suggest that the underlying mechanisms of enhancement of FER under acute stress are not considerably affected in relation to overall FER performance. Therefore, we argue that enhanced detection of emotional cues after stress may be a valid adaptive response in individuals with schizophrenia spectrum disorder. An increased sensitivity under acute stress to social cues may help individuals detect potential threats or sources of social support in their social environment. Individuals with schizophrenia spectrum disorder show general impairments in FER, but a better sub-task naming performance under acute psychosocial stress.

Our findings of higher baseline cortisol levels and blunted cortisol reactivity in the *PAT* group, likely related to higher stress susceptibility and HPA dysregulation, are consistent with the study hypothesis and findings from other studies (e.g., 9, 10, 62–64).

Although the rise of subjective perception of momentary strain consequent upon the induction of psychosocial stress in our paradigm was significant in both groups, it is worth noting that the pre-stress level of subjective strain was already higher in the *PAT* group, most probably due to illness chronicity (11). In parallel to the observation of blunted cortisol responses, subjective strain did not increase as profoundly in the *PAT* group as in the controls, resulting in similar peak levels. It is possible that completing study questionnaires influenced emotional arousal, although the results of the questionnaires may suggest similar emotional arousal in the *CTL* and *PAT* groups.

As hypothesised, non-social cognition, assessed using an arithmetic task as a proxy measure, was impaired in the *PAT* group as compared to the *CTL* group. This finding is unsurprising as cognitive impairments have long been accepted as core features of schizophrenia (65). Nevertheless, the observed impairments in FER tasks underline the importance of social skills training in schizophrenia spectrum disorders. Literature points to the importance of coping and adequate social interactions to lower a possible elevated risk for violence in stressful situations (28), which is a frequent treatment target in forensic settings.

### 4.1 Limitations

We selected an all-male sample to minimise a potential sex bias in FER, which limits the generalisability of our results to female individuals with schizophrenia spectrum disorder. The *PAT* group comprised people with chronic forms of schizophrenia, which may have different stress susceptibility and higher impairments in FER compared to those with first-episode psychoses. Due to a lack of adequate statistical power (i.e., a small sample size), we could not investigate the possible impacts of individual parental bonding styles on FER. Additionally, the design of the study paradigm may have introduced bias by including CTQ and PBI before testing. It is possible that emotional arousal to questions may have influenced facial emotion interpretation. Furthermore, the *PAT* group had mild symptomatology and relatively high IQ, which limits the generalisability of our findings to individuals with schizophrenia who may exhibit a higher psychopathological load and lower IQs. Moreover, the *PAT* group received antipsychotic medication, which may impair cognitive functioning. This possible confounder was not controlled for in the present study. Given that the sample did not include individuals with schizophrenia without a history of violence, no inferences about the direct influence of FER on violence in schizophrenia can be drawn.

### 4.2 Conclusion

In sum, the present study extends previous findings regarding the cognitive effects of stress on FER in the context of social cognitive functioning. The results confirmed higher baseline cortisol levels and momentary strain before stress induction in individuals with schizophrenia than in controls. A significant increase in cortisol levels was recorded in controls only after stress induction.

Our results also demonstrated that acute psychosocial stress increased FER performance in the sub-task of naming emotions and induced a non-significant performance change in the face comparison task in individuals with schizophrenia spectrum disorder in a forensic setting. In line with findings from previous studies, arithmetic task performance was worse in the *PAT* group.

In this study, stress levels (both subjectively and physiologically) were higher in individuals with schizophrenia spectrum disorders before stress induction than controls. Previous findings that cortisol response to psychosocial stress is blunted in schizophrenia spectrum disorder were also confirmed by our results.

However, underlying mechanisms of enhancement of FER under acute stress were not considerably affected in individuals with schizophrenia compared to healthy controls. This heightened sensitivity may help individuals adapt to social stress and modulate more complex social behaviour and decision-making. Lastly, our results reproduce findings of cognitive impairments in FER and non-social arithmetic tasks in individuals chronically affected by schizophrenia spectrum disorder. More research is needed to determine the impact of these findings on real-world, contextually rich social interactions. Future investigations should be initiated to control for the influence of different parental bonding styles on FER in control and patient groups.

## Data availability statement

The data is not publicly available for legal reasons. Requests to access the datasets should be directed to the corresponding author.

## Ethics statement

The studies involving humans were approved by the Ethics Committee of Northwestern and Central Switzerland (EKNZ Reference number 2016–00701). The studies were conducted in accordance with the local legislation and institutional requirements. The participants provided their written informed consent to participate in this study.

## Author contributions

HH: Writing – review & editing, Writing – original draft, Visualization, Supervision, Resources, Project administration, Methodology, Investigation, Funding acquisition, Conceptualization. GD: Writing – review & editing, Writing – original draft, Visualization, Validation, Software, Resources, Project administration, Investigation, Formal analysis, Data curation. MG: Writing – review & editing, Writing – original draft. TV: Writing – review & editing, Writing – original draft, Supervision, Resources, Investigation.
