# Supplementary material for: Impact of psychosocial stress on facial emotion recognition in schizophrenia and controls: an experimental study in a forensic sample
Source: Front Psychiatry. 2024 Jul 16;15:1358291. doi: 10.3389/fpsyt.2024.1358291 (PMC11287427; doi:10.3389/fpsyt.2024.1358291)
Supplement: Supplementary file 1 [file DataSheet_1.docx]

**Supplementary Material**

# Analysis of Deviance Tables

**Table S1.** Results of the Wald-type Χ² analysis of deviance of the repeated measures LMMs and the one-way ANOVA of arithmetic task performance.

| **Outcome Measure** | **Effect** | **Χ²_(1)_^**^** | ***P*** | **ω_p_²^****^** |
| --- | --- | --- | --- | --- |
| Salivary Cortisol | (Intercept) | 114.56 | < .001 |  |
| (nmol/L) | *Group* | 6.76 | .009 | 0.16 |
|  | *Stress* | 13.93 | < .001 | 0.33 |
|  | *Group* × *Stress* | 4.90 | .03 | 0.13 |
|  |  |  |  |  |
| KAB rating of momentary strain | (Intercept) | 341.57 | < .001 |  |
| KAB Score (1 – 6) | *Group* | 2.83 | .09 | 0.05 |
|  | *Stress* | 80.04 | < .001 | 0.71 |
|  | *Group* × *Stress* | 7.30 | .007 | 0.16 |
|  |  |  |  |  |
| FER A, naming emotions | (Intercept) | 526.33 | < .001 |  |
| Hit Rate (%) | *Group* | 12.58 | < .001 | 0.26 |
|  | *Stress* | 7.26 | .007 | 0.16 |
|  |  |  |  |  |
| FER B, comparing emotional faces | (Intercept) | 378.03 | < .001 |  |
| Hit Rate (%) | *Group* | 14.12 | < .001 | 0.28 |
|  |  |  |  |  |
| **Outcome Measure** | **Effect** | ***F* ^***^** | ***P*** | **ω²** |
| Arithmetic Task, non-social cognition | (Intercept) | 544.04 | < .001 |  |
| Hit Rate (%) | *Group* | 1.58 | .002 | 0.25 |
|  |  |  |  |  |

*Note.* * rank-biserial correlation coefficient as measure of effect size;
** Χ² with 1 degree of freedom
***ANOVA F value with degrees of freedom (1, 30)
**** effect size – small ω^2^ ≥ .01, medium: ω^2^ ≥ .06, large: ω^2^ ≥ .14

# Contrasts Tables

**Table S2.** Contrast estimates for simple main effects and effect sizes for all outcome measures. In the presence of interactions, the simple effects of a factor at each level of the second factor were estimated. Estimates, standard errors, and confidence limits are in the Box-Cox transformed scale.

|  |  |  |  |  |  |  |  |  |  |  |  |
| --- | --- | --- | --- | --- | --- | --- | --- | --- | --- | --- | --- |
| **Outcome Measure** | **(Simple) Main Effect** | **Contrast** | **2^nd^ Factor Level** | **Estimate** | ***SE*** | ***DF*** | **95 % *CI*** | | ***T*** | ***P*** | **Hedges’ *g*** |
|  |  |  |  |  |  |  | **Lower** | **Upper** |  |  |  |
| Salivary Cortisol, λ = 0.20 | *Group* | *CTL - PAT* | *pre* | -1.27 | 0.34 | 40.33 | -1.96 | -0.58 | -3.70 | .001 | -1.32 |
|  | *Group* | *CTL - PAT* | *post* | -0.48 | 0.41 | 39.05 | -1.32 | 0.36 | -1.16 | .25 | -0.41 |
|  | *Stress* | *pre - post* | *CTL* | -1.06 | 0.28 | 26.35 | -1.64 | -0.47 | -3.71 | .001 | -0.97 |
|  | *Stress* | *pre - post* | *PAT* | -0.27 | 0.21 | 24.11 | -0.71 | 0.17 | -1.27 | .22 | -0.26 |
|  |  |  |  |  |  |  |  |  |  |  |  |
| KAB Score, λ = 0.06 | *Group* | *CTL - PAT* | *pre* | -0.58 | 0.20 | 53.57 | -0.98 | -0.17 | -2.85 | .006 | -0.98 |
|  | *Group* | *CTL - PAT* | *post* | 0.03 | 0.19 | 53.57 | -0.35 | 0.42 | 0.17 | .87 | 0.06 |
|  | *Stress* | *pre - post* | *CTL* | -1.32 | 0.14 | 30.00 | -1.61 | -1.03 | -9.30 | < .001 | -2.35 |
|  | *Stress* | *pre - post* | *PAT* | -0.71 | 0.18 | 30.00 | -1.07 | -0.35 | -4.01 | < .001 | -1.19 |
|  |  |  |  |  |  |  |  |  |  |  |  |
| FER A Hit Rate, λ = 2.28 | *Group* | *CTL - PAT* |  | 0.08 | 0.02 | 31.00 | 0.03 | 0.12 | 3.55 | .001 | 0.86 |
|  | *Stress* | *pre - post* |  | -0.03 | 0.01 | 32.00 | -0.05 | -0.01 | -2.69 | .01 | -0.41 |
|  |  |  |  |  |  |  |  |  |  |  |  |
| FER B Hit Rate, λ = 3.03 | *Group* | *CTL - PAT* |  | 0.07 | 0.02 | 31.00 | 0.03 | 0.11 | 3.76 | < .001 | 0.91 |
|  |  |  |  |  |  |  |  |  |  |  |  |
| AT Hit Probability, λ = 1 | *Group* | *CTL - PAT* |  | 0.11 | 0.03 | 30.00 | 0.04 | 0.17 | 3.40 | < .001 | 1.17 |
|  |  |  |  |  |  |  |  |  |  |  |  |

Note. *SE*: standard error; *DF*: degrees of freedom; *CI*: confidence interval.

# Estimated Marginal Means Table

**Table S3.** Estimated marginal (EM) means for all presented analysis in the response scale.

| **Outcome Measure** | ***Group*** | ***Stress*** | **EM Mean** | **95 % CI** | |
| --- | --- | --- | --- | --- | --- |
|  |  |  |  | **Lower** | **Upper** |
| Salivary Cortisol | *CTL* | *pre* | 2.16 | 1.38 | 3.27 |
| (nmol/L) | *PAT* | *pre* | 5.78 | 4.09 | 7.98 |
|  | *CTL* | *post* | 4.97 | 3.07 | 7.70 |
|  | *PAT* | *post* | 6.96 | 4.72 | 10.00 |
|  |  |  |  |  |  |
| KAB Score | *CTL* | *pre* | 1.80 | 1.52 | 2.09 |
| (1 – 6) | *PAT* | *pre* | 2.59 | 2.11 | 3.10 |
|  | *CTL* | *post* | 3.76 | 3.24 | 4.30 |
|  | *PAT* | *post* | 3.70 | 3.33 | 4.09 |
|  |  |  |  |  |  |
| FER A | *CTL* |  | 75.79 | 71.99 | 79.36 |
| Hit Rate (%) | *PAT* |  | 63.90 | 57.31 | 69.71 |
|  |  | *pre* | 67.87 | 63.74 | 71.71 |
|  |  | *post* | 72.37 | 68.69 | 75.82 |
|  |  |  |  |  |  |
| FER B | *CTL* |  | 82.84 | 79.63 | 85.81 |
| Hit Rate (%) | *PAT* |  | 71.17 | 64.39 | 76.85 |
|  |  |  |  |  |  |
| Arithmetic Task | *CTL* |  | 42.02 | 37.48 | 46.56 |
| Hit Rate (%) | *PAT* |  | 31.32 | 26.78 | 35.86 |
|  |  |  |  |  |  |

Note. *CI*: confidence interval.

# Salivary Cortisol Measurement

The cortisol saliva samples were processed at the in-house laboratory of the University Psychiatric Clinics in Basel. Prior to cortisol measurement, each saliva sample underwent vortexing and centrifugation at 1500 x g for 15 minutes to eliminate any precipitate. Cortisol concentrations were quantified using a high-sensitivity salivary cortisol enzyme immunoassay kit (Salimetrics Europe, UK), following the manufacturer’s protocol. Optical density readings were obtained at 450 nm using the Cytation 3 Cell Imaging Multimode Reader (BioTek). The cortisol ELISA demonstrated a sensitivity of <0.007 µg/dL, with an intra-assay coefficient of variation (CV) of 4.6% and an inter-assay CV of 6%.

# Power Analysis

An a priori power analysis to determine the study’s adequate sample size was performed using the G-Power application for Microsoft Windows. Due to the absence of previous research, the effect size expectations were guided by Cohen’s (1988) effect size limits and generally apply to all variables that were analyzed using LMM. It can be safely assumed that LMMs offer not less statistical power than repeated measures analysis of variance (RMANOVA), thus the power analysis was based on RMANOVA for group by repeated measure interactions with standard a priori parameter settings for finding at least medium sized effects with *f* ≥ 0.25, aiming at a maximal Type II error probability of β = .20 (power = 0.8) and a Type I error probability of α = .05. Finding small effect sizes with *f* < 0.25 was disregarded knowingly. The result was a total sample size of *N* = 34 (17 per group) satisfying these constraints. The same calculation for the repeated measures (within) main effect yielded the same result. A sensitivity calculation with these same constraints showed that a projected sample size of *N* = 34 would suffice to detect large (between) group differences with an effect size of *f* ≥ 0.43 / ω^2^ ≥ 0.16. The final sample size became reduced to 32 due to two dropouts; one in each group. A sensitivity analysis for *N* = 32 indicated only a negligible loss, increasing the minimal detectable within/within-between interaction effect size to *f* ≥ 0.256 / ω^2^ ≥ 0.062, respectively the group effect size to *f* ≥ 0.443 / ω² ≥ 0.164, while maintaining a power of 0.8.

The one-way ANOVA that was performed to analyse the group difference in the arithmetic task was a secondary analysis and was not considered in the sample size calculation. To detect a medium sized effect of Cohen's f of 0.25 or higher using a one-way ANOVA, a sample size of *N* = 128 would have been necessary, which was not feasible. The measured group effect turned out to be quite large (*f* = 0.58, *omega*² = 0.25, *d* = 1.24, latter from *F*-value), and when we compared the point estimates and standard errors obtained through calculation to their bootstrapped counterparts, they showed only minimal discrepancy (< 0.35 %), which may support confidence in our finding.

# Complete List of used Karolinska Directed Emotional Faces

| AF01AFS | AF01ANS | AF01DIS | AF01HAS | AF01SAS | AF02AFS |
| --- | --- | --- | --- | --- | --- |
| AF02ANS | AF02DIS | AF02HAS | AF02SAS | AF03AFS | AF03ANS |
| AF03DIS | AF03HAS | AF03SAS | AF04ANS | AF04SAS | AF05AFS |
| AF05DIS | AF06HAS | AF07AFS | AF07ANS | AF07DIS | AF09HAS |
| AF09SAS | AF10AFS | AF10DIS | AF10HAS | AF11AFS | AF11ANS |
| AF11DIS | AF11HAS | AF11SAS | AF12ANS | AF12DIS | AF13AFS |
| AF13HAS | AF13SAS | AF14ANS | AF14DIS | AF14HAS | AF14SAS |
| AF15DIS | AF15HAS | AF15SAS | AF16AFS | AF16ANS | AF16HAS |
| AF17AFS | AF17ANS | AF17DIS | AF17HAS | AF17SAS | AF19DIS |
| AF19HAS | AF19SAS | AF20AFS | AF20SAS | AF21HAS | AF22SAS |
| AF23AFS | AF23ANS | AF23DIS | AF23HAS | AF23SAS | AF24SAS |
| AF25DIS | AF26HAS | AF27ANS | AF29DIS | AF30ANS | AF30HAS |
| AF31DIS | AF32AFS | AF32HAS | AF33AFS | AF33ANS | AF34AFS |
| AM01AFS | AM01ANS | AM01DIS | AM01HAS | AM01SAS | AM02AFS |
| AM02ANS | AM02DIS | AM02HAS | AM02SAS | AM04AFS | AM04ANS |
| AM04DIS | AM04HAS | AM04SAS | AM05AFS | AM05ANS | AM05DIS |
| AM05HAS | AM05SAS | AM06AFS | AM06SAS | AM07AFS | AM07DIS |
| AM07SAS | AM08ANS | AM08HAS | AM09AFS | AM09DIS | AM09HAS |
| AM10AFS | AM10ANS | AM10DIS | AM10HAS | AM10SAS | AM11DIS |
| AM11HAS | AM11SAS | AM13AFS | AM13ANS | AM13DIS | AM13SAS |
| AM14ANS | AM14DIS | AM14SAS | AM17HAS | AM18AFS | AM18DIS |
| AM18HAS | AM21HAS | AM21SAS | AM22AFS | AM22ANS | AM22DIS |
| AM22HAS | AM22SAS | AM23AFS | AM23DIS | AM23HAS | AM24DIS |
| AM25AFS | AM25ANS | AM25SAS | AM27AFS | AM27ANS | AM27DIS |
| AM27HAS | AM27SAS | AM29DIS | AM29HAS | AM29SAS | AM31AFS |
| AM31ANS | AM32HAS | AM32SAS | AM33ANS | AM33SAS | AM34AFS |
| AM34ANS | AM34DIS | AM34HAS | AM34SAS | AM35ANS | AM35DIS |
